# Announcing the *mSystems* special collection on microbial dormancy

**DOI:** 10.1128/msystems.01005-24

**Published:** 2024-09-12

**Authors:** Ashley Shade

**Affiliations:** 1 Universite Claude Bernard Lyon 1, CNRS, INRAE, VetAgro Sup, Laboratoire d'Ecologie Microbienne LEM, CNRS UMR5557, INRAE UMR1418, Villeurbanne, France; University of California San Diego, La Jolla, California, USA

**Keywords:** antimicrobial activity, dormancy, persister cells, stress response, microbiome, evolution, ecology

## EDITORIAL

Many microorganisms can persist in a reversible state of reduced metabolic activity called dormancy. Dormancy allows microbes to survive unfavorable conditions, disperse and immigrate widely, broaden community diversity, extend lifespan, and preserve genetic information for future generations. Yet, there remain critical and fundamental unknowns about dormancy and its implications for microbial systems biology.

*mSystems* welcomes original Research Articles, Methods and Protocols, Opinions/Hypotheses, or Perspective articles that advance understanding of microbial dormancy and its consequences for population or community ecology, evolution, microbe-microbe or host-microbial interactions, and system metabolism or cellular processes. Topics of interest include but are not limited to the following:

1. Persister cells and antimicrobial, metal, or other abiotic or biotic control of microbial activity switching.
2. Viable but non-cultivable phenotypes, their drivers, and their consequences for cellular systems, community ecology, or population fitness.
3. New methods for discriminating activity states among microbial cells, including laboratory and computational/bioinformatic tools.
4. Assessment of microbial activity (e.g., using SIP, BONCAT, flow cytometry, and 16S rRNA ratios) and to inform understanding of patterns of biodiversity, ecology, or evolution.
5. Phylogenetic insights into the distributions and diversification of dormancy strategies.
6. Implications of microbial dormancy for microbe-microbe or microbe-host interactions (including competition, synergisms, symbioses, or microbiome outcomes).
7. Dormancy-driven alterations in cellular, population, or microbiome primary or secondary metabolism, metabolic networks, or regulation.
8. Development of theory, conceptual frameworks, or mathematical models for understanding dormancy or activity state switching or predicting dormant states.
9. Discovery of new biological signatures of dormant states.

Articles belonging to the special collection will be published as they are accepted, and we especially welcome the application of multidisciplinary and innovative approaches to understanding the drivers and consequences of dormancy in microbial systems.

This special collection is catalyzed in parallel to the *mSystems* Thinking Series webinars, “Critical Concepts in Dormancy,” which will be held on 17 September 2024 and 7 November 2024.

